# The Impact of Age and Sex on Mouse Models of Melioidosis

**DOI:** 10.3390/pathogens9020113

**Published:** 2020-02-11

**Authors:** Christopher P. Klimko, Sylvia R. Treviño, Alicia M. Moreau, Michael J. Aponte Cuadrado, Joshua R. Meyer, David P. Fetterer, Susan L. Welkos, Patricia L. Worsham, Norman Kreiselmeier, Carl Soffler, Christopher K. Cote

**Affiliations:** 1 United States Army Medical Research Institute of Infectious Diseases (USAMRIID), Bacteriology Division 1425 Porter Street, Fort Detrick, Frederick, MD 21702, USA; christopher.p.klimko2.ctr@mail.mil (C.P.K.); sylvia.r.trevino.civ@mail.mil (S.R.T.); michael.j.apontecudrado.mil@mail.mil (M.J.A.C.); joshua.r.meyer6.mil@mail.mil (J.R.M.); susan.l.welkos.civ@mail.mil (S.L.W.); patricia.l.worsham.civ@mail.mil (P.L.W.); carl.soffler.mil@mail.mil (C.S.); 2 United States Army Medical Research Institute of Infectious Diseases (USAMRIID), Pathology Division 1425 Porter Street, Fort Detrick, Frederick, MD 21702, USA; alicia.m.moreau.mil@mail.mil (A.M.M.); norman.kreiselmeier.mil@mail.mil (N.K.); 3 United States Army Medical Research Institute of Infectious Diseases (USAMRIID), Biostatistics Division 1425 Porter Street, Fort Detrick, Frederick, MD 21702, USA; david.p.fetterer.ctr@mail.mil

**Keywords:** *Burkholderia pseudomallei*, melioidosis, mice, pathology, median lethal dose, inhalational, intraperitoneal

## Abstract

Mouse models have been used to generate critical data for many infectious diseases. In the case of *Burkholderia pseudomallei*, mouse models have been invaluable for bacterial pathogenesis studies as well as for testing novel medical countermeasures including both vaccines and therapeutics. Mouse models of melioidosis have also provided a possible way forward to better understand the chronicity associated with this infection, as it appears that BALB/c mice develop an acute infection with *B. pseudomallei*, whereas the C57BL/6 model is potentially more suggestive of a chronic infection. Several unanswered questions, however, persist around this model. In particular, little attention has been paid to the effect of age or sex on the disease outcome in these animal models. In this report, we determined the LD_50_ of the *B. pseudomallei* K96243 strain in both female and male BALB/c and C57BL/6 mice in three distinct age groups. Our data demonstrated a modest increase in susceptibility associated with sex in this model, and we documented important histopathological differences associated with the reproductive systems of each sex. There was a statistically significant inverse correlation between age and susceptibility. The older mice, in most cases, were more susceptible to the infection. Additionally, our retrospective analyses suggested that the impact of animal supplier on disease outcome in mice may be minimal. These observations were consistent regardless of whether the mice were injected with bacteria intraperitoneally or if they were exposed to aerosolized bacteria. All of these factors should be considered when designing experiments using mouse models of melioidosis.

## 1. Introduction

*Burkholderia pseudomallei*, causing melioidosis, is a Gram negative bacterium designated a Tier 1 select agent by both the U.S. Department of Health and Human Services and U.S. Department of Agriculture. This bacterium is endemic in Southeast Asia and northern Australia, but recent work has clearly demonstrated that it is quickly becoming a global concern [1,2,3,4]. *B. pseudomallei* can infect humans and animals via several routes of infection (i.e., inhalation of aerosolized bacteria, ingestion of contaminated drinking water, or through inoculation of a subcutaneous lesion) [5,6,7,8,9]. Inhalational melioidosis has been associated with monsoonal rains in endemic areas and is also a primary concern in the biodefense research community [10,11]. Pneumonia is a common presentation, which can be caused by a primary introduction of aerosolized bacteria into the lungs or a secondary pneumonia resulting from hematogenous spread to the lungs after a primary infection elsewhere [12,13]. There are currently no approved vaccines for *B. pseudomallei* and antibiotic treatment can be hampered by non-specific symptomology, the protracted two-phase course of treatment required, and the high rate of naturally occurring antibiotic-resistant strains [14,15]. Because of the public health and biodefense concerns associated with this bacterium, efforts are underway to develop effective medical countermeasures [15,16]. Accordingly, appropriate and well-characterized animal models are required for the development and testing of such efforts.

The mouse model has afforded many research groups the opportunity to characterize the pathogenesis of *B. pseudomallei*. It has been reported that BALB/c mice may represent an acute model of melioidosis, whereas C57BL/6 mice are more resistant to the bacterium and may provide a more chronic model of the disease [17,18,19,20,21]. While there is some debate about the definition of chronicity in a mouse model, there are clear differences in susceptibilities, dissemination patterns, and resulting immune responses observed in these mice after exposure to *B. pseudomallei* [22,23,24]. Experiments conducted with either BALB/c or C57BL/6 mice have provided the main body of knowledge available for *B. pseudomallei* mouse models. Various routes of exposure have been used to model disease progression in mice [17]. Intraperitoneal (IP) inoculation has been demonstrated to be an effective route of infection for characterizing pathogenesis and comparing the virulence of human clinical isolates of *B. pseudomallei* [22,25,26,27]. The IP route provides a means for laboratories to readily compare testing and evaluation data because it is easy to accomplish with fewer confounding variables when compared to the inhalational route. By using the IP route of exposure, Welkos et al. demonstrated significant differences in virulence amongst a panel of clinical isolates [25]. In this report, it was also established that mice should be monitored for a prolonged period of time after infection by this route to truly ascertain the effectiveness of a vaccine or therapeutic. Accordingly, LD_50_ values were established for Day 21 and Day 60 post infection [25]. These analyses also led to unique strain-characterization criteria (i.e., some strains were more acutely virulent, whereas other strains required additional time to cause similar lethality). 

Other reports have also used the IP model of inoculation. Dannenberg and Scott infected Albino Namru mice via the IP route to look at the cellular response to the ensuing infection with a mouse-adapted strain of *B. pseudomallei* [28]. Tan et al. utilized IP infections in order to characterize the resulting immune response to the acute phase of the infection using both C57BL/6 and BALB/c mice [23]. While the IP route is obviously not a natural route of infection, it has proven to be a useful tool for *B. pseudomallei* research, and the resulting disease progression presents with interesting pathology and clinical signs which can be anecdotally compared to human case reports. An important aspect of this model is the fact that the caudal half of the animal is substantially impacted by the disease, often resulting in rear-leg paralysis and other physical manifestations. The pyogranulomas formed as a result of the *B. pseudomallei* infection can impact joints, muscle, bone, and nerves and the resulting paralysis has been used as an early endpoint criterion for euthanasia [22,25]. Human case reports have documented similar observations [29,30]. Even with this route’s associated sequelae, the IP model will continue to be an important tool.

Inhalational melioidosis can be initiated after *B. pseudomallei* bacteria become aerosolized [10,11,31]. This route of exposure has been studied using both C57BL/6 and BALB/c mice [22,23,28,32,33]. Recent reports have detailed that differences between bacterial strains can be observed after exposure to aerosolized bacteria [24,33]. The IP model offers large ranges of determined LD_50_ values between clinical isolates tested [25,34]; however, the differences observed in calculated LD_50_ values when mice are exposed to aerosolized *B. pseudomallei* are less appreciable. Exposure to aerosolized bacteria causes a primary pneumonia that results in a highly disseminated disease characterized by pyogranuloma formation [22,28,33]. The pathologies observed after being exposed to aerosolized bacteria have some similarities to that seen after IP inoculation, but the variety and severity of these observations are different from those observed after following the IP route [22]. For example, caudal disease progression (e.g., rear-end paralysis or tail lesions) has been observed after mice are exposed to aerosolized *B. pseudomallei* bacteria, but this is far less commonly observed and tends be less severe when compared to mice receiving the bacteria via an IP injection. These differences have been hypothesized to be due to location of initial site of entry and proximity to draining lymph nodes. Regardless of the mouse strain, route of exposure, or the bacterial isolate, melioidosis in the mouse model is heterogeneous in regards to clinical signs and disease outcome [17,22,25,35].

Key factors of the mouse model, such as age and sex of the animals, have not been fully evaluated. The sex of the mice is of particular interest [36]. The majority of melioidosis cases in humans have been reported in male patients [37,38,39]. While this sex bias observed in melioidosis may be primarily associated with health and/or occupational risk factors (e.g., alcoholism or occupational exposure through rice cultivation), it is important to examine both sexes of mice in order to get the most complete dataset possible [40]. There have been several reports using male mice as melioidosis models [41,42,43,44,45,46,47,48,49,50,51], but only a few have actually either compared males and females or characterized the disease pathology within male mice [41,52,53,54]. Emery et al. reported evidence demonstrating that female mice in their experiments tended to be more resistant to infection caused by intranasal instillation of *B. pseudomallei* [53]. Additionally, age of experimental animals is an important variable for infectious disease research [55,56]. Many aspects of the immune response are altered with age and these alterations have been demonstrated to result in differential results upon both viral and bacterial infection [57,58,59,60,61]. The age of mice used in melioidosis research may be important for several reasons. Melioidosis is known to re-emerge or relapse in individuals who had become infected (some asymptomatically) years or decades earlier [62,63]. It has also been suggested that older individuals may be more likely to be exposed to occupational hazards, since much of the younger population are moving towards urban centers in Southeast Asia [64]. When dealing with the development of novel medical countermeasures, age is also an important parameter. For example, when testing and evaluating a vaccine, the mice will likely be at least several months older after the vaccine regimen is completed. Thus, it is uncertain how these older mice would survive the challenge dose or if a significantly different median lethal dose would be expected.

Lastly, we compared these current data with historical data to determine whether source vendor had any significant impact on disease outcome. There are many factors that impact the utility of mice for infectious disease research. These include husbandry conditions, diet, genetic drift of colony, and overall health [65,66,67]. Historically, we have observed differences between mice received from different locations after exposure to bacteria which cause acute infections and we wanted to document any such observations with the melioidosis mouse model. The objectives of the work described here were to determine the median lethal doses of *B. pseudomallei* K96243 in both BALB/c and C57BL/6 mice of both sexes at distinct age points. These data are important to ensure thorough downstream evaluations of novel medical countermeasures against melioidosis.

## 2. Results

### 2.1. The Impact of Sex on the Disease Outcome in the Mouse Model of B. pseudomallei Infection

**Figure 1:**
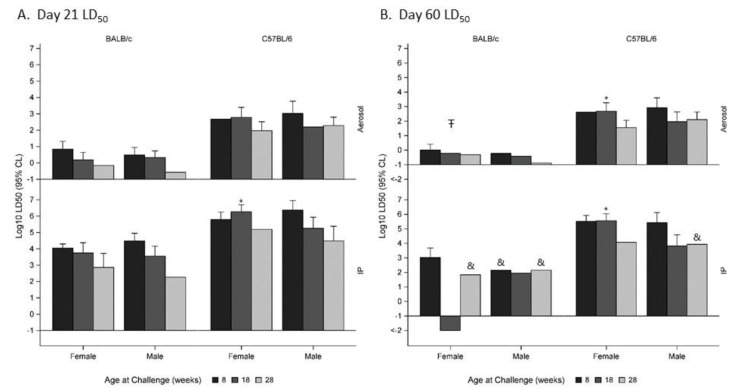
Impact of sex on susceptibility to *Burkholderia pseudomallei* 21 days (**A**) and 60 days (**B**) post challenge. Where an unbounded confidence interval was found, the confidence interval has been omitted. (Ŧ *p* < 0.05 compared to males, pooling all age groups. * *p* < 0.05 compared to age-matched male mice. & No survivors remained, the least challenge dose is plotted).

In this report, we performed head-to-head comparisons of age- and sex-matched mice, and the calculated median lethal doses for each group are described in Table 1. In some cases, the trends from these data suggested that female mice may have been more resistant to the infection when compared to their male counterparts. However, there were no statistically significant impacts of sex on disease outcome in the BALB/c mouse model (*p* > 0.05) when examining data through Day 21 post-infection (Figure 1A). These data resulted in an overall female LD_50_: male LD_50_ ratio of nearly 1.0. This was the case regardless of the age of mice at challenge or route of exposure. There was a statistically significant difference (*p* < 0.05) when examining the impact of sex on the disease outcome when C57BL/6 mice aged 18 weeks were exposed IP to *B. pseudomallei* (Figure 1A). However, the effect of sex was not significant among other strata of the Day 21 data. The impact of sex was more pronounced when examining Day 60 LD_50_ estimates (Figure 1B). In these comparisons, statistically significant sex-biased effects were observed among both BALB/c and C57BL/6 mice, with females having a greater LD_50_ than age- and strain-matched males (*p* < 0.05). The effect was greatest among animals aged 18 weeks at the time of initial exposure.

### 2.2. The Impact of Age on the Disease Outcome in the Mouse Model of B. pseudomallei Infection 

**Figure 2:**
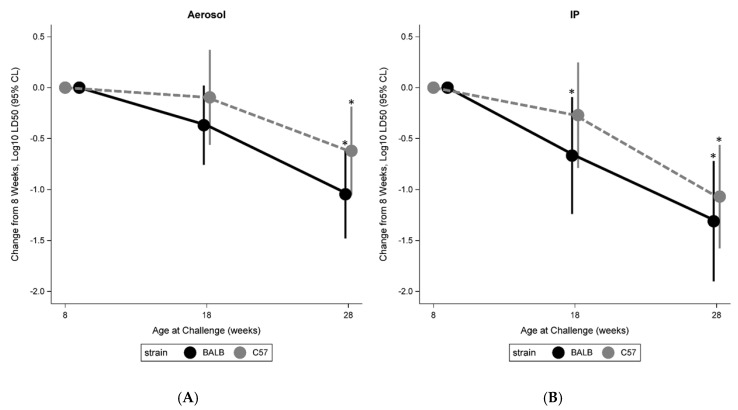
Impact of age on susceptibility to *B. pseudomallei* 21 days post infection by (**A**) aerosol and (**B**) IP routes. (* *p* < 0.05 compared to mice aged 8 weeks.).

The effects of age on Day 21 LD_50_ were similar in the IP and aerosol exposure routes (Figure 2). Among BALB/c mice, statistically significant (*p* < 0.05) reductions in the LD_50_ relative to 8 weeks were observed in mice aged 28 weeks, with reductions of 1.0 and 1.3 Log_10_ CFU in the Aerosol and IP groups, respectively. Among C57BL/6 mice, significant reductions were also observed at 28 weeks, with reductions of 0.6 and 1.1 Log_10_ CFU in the Aerosol and IP groups, respectively. Among mice aged 18 weeks, the reduction from 8 weeks was significant only in BALB/c mice exposed via the IP route. However, the observed reductions in LD_50_ in all groups were consistent with the overall trend toward increased susceptibility in aged mice. Similar trends were observed when examining the Day 60 LD_50_ (data not shown).

### 2.3. The Impact of Animal Supplier on the Disease Outcome in the Mouse Model of B. pseudomallei Infection

We performed retrospective analyses using several permutations to evaluate the impact that vendor source had on LD_50_. It was determined that overall, source vendor played little to no role in disease outcome. As depicted in Table 2, there was only a significant difference between 8 week old female C57BL/6 mice acquired from Envigo or Charles River challenged by the IP route (*p* = 0.037). While these limited retrospective analyses offer only a glimpse into source vendor as a parameter, these data suggest that mice from different vendors can provide relatively reproducible data.

### 2.4. Sex-Specific Pathology Associated with B. pseudomallei Infection in Mice

While the main objective of these studies was to determine age- and sex-associated differences in susceptibility to *B. pseudomallei*, we also documented the sex-specific pathology observed in these mice that met early endpoint euthanasia criteria.

#### 2.4.1. Intraperitoneal Route of Infection

**Figure 3:**
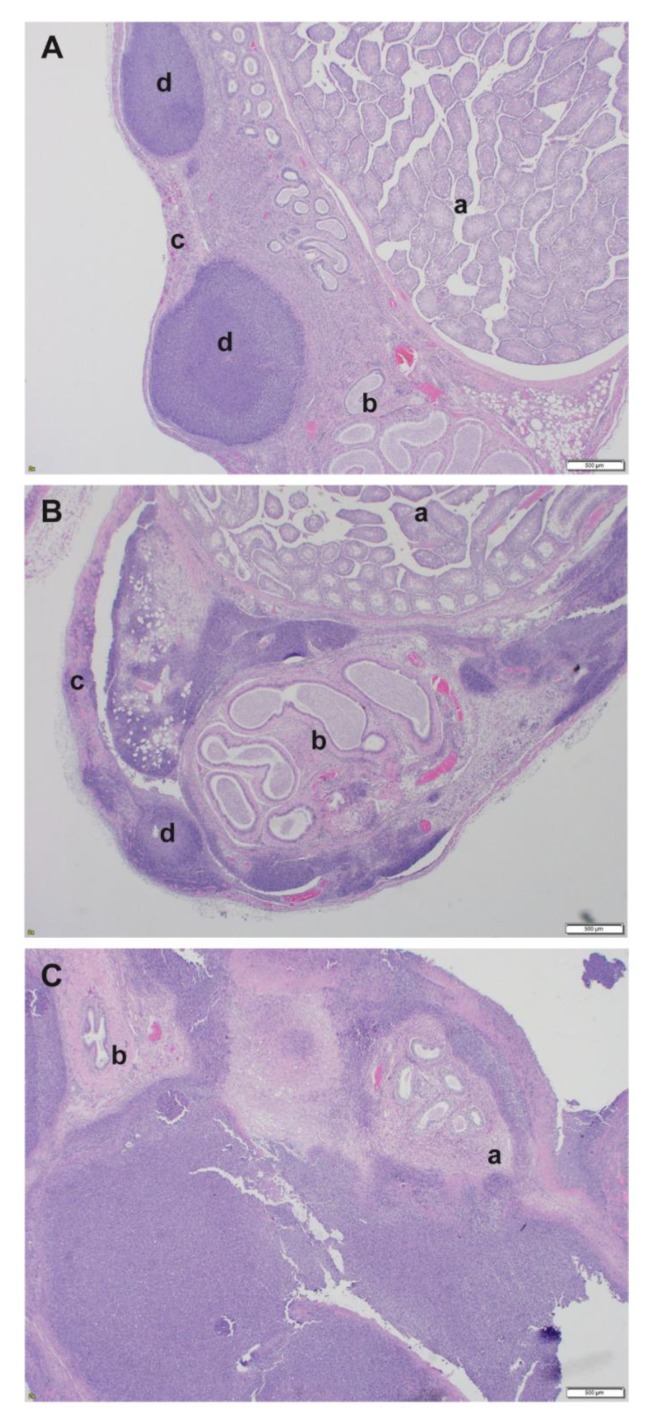
Male reproductive tract pathology associated with intraperitoneal exposure to *B. pseudomallei*. (**A**) Spermatic cord exhibiting mild pyogranulomatous inflammation with necrosis and abscesses (BALB/c mouse 18 weeks at challenge, H&E); a: seminiferous tubules, b: epididymis, c: spermatic cord, d: abscess. (**B**) Spermatic cord exhibiting moderate pyogranulomatous inflammation with necrosis and abscess. Epididymis with mild pyogranulomatous inflammation with necrosis; Testes, tunica albuginea with mild pyogranulomatous inflammation and necrosis (BALB/c mouse 28 weeks at challenge, H&E); a: seminiferous tubules, b: epididymis, c: spermatic cord, d: abscess (**C**) Testes with severe pyogranulomatous inflammation, necrosis, and abscesses (BALB/c mouse 28 weeks at challenge, H&E); a: epididymis, b: vas deferens.

The most significant pathological lesions in mice infected via intraperitoneal route were present throughout the peritoneum and on serosal surfaces of the reproductive tracts, with extension into the organ as lesions progressed. Mild to moderate peritonitis was present in 16/21 (76%) BALB/c males with all age groups affected. A mild to marked peritonitis was present in 18/22 (82%) BALB/c females with all age groups affected. Mild to moderate peritonitis was present in 10/19 (53%) C57BL/6 males and in 7/13 (54%) C57BL/6 females, with all age groups affected. The segment of the reproductive tract that was most significantly affected in male BALB/c mice that were infected via IP route was the spermatic cord and tunic of the epididymis. All three age groups (100% of male BALB/c mice) had minimal to severe inflammation in the spermatic cord and/or epididymis. There was one male mouse that had minimal inflammation and one male mouse that had mild inflammation in the spermatic cord, but the remaining 19/21 (90%) had moderate to marked inflammation. Additionally, 85%–100% of all male BALB/c had necrosis and abscesses within the spermatic cord and/or epididymis (Figure 3A,B). In 2/10 (20%) 28 week old challenged mice, the marked to severe inflammation and/or abscesses unilaterally effaced the epididymis and seminiferous tubules of the testes (see Figure 3C). There were a variety of additional lesions noted in the testes and accessory sex glands. Mild to marked pyogranulomatous inflammation in the tunica albuginea (capsule) of the testes was noted in 18/21 (86%) BALB/c males. Minimal to marked inflammation from the testicular capsule extended into the interstitium of the testes in 12/21 (57%), resulting in degeneration and necrosis of the seminiferous tubules in 10/21 (48%) BALB/c males. There was mild inflammation in the prostate of 5/21 (24%) BALB/c males, mild to moderate inflammation in the coagulating gland of 3/21 (14%) BALB/c males, and mild to moderate inflammation in the seminal vesicles of 2/21 (10%) BALB/c males.

Similar lesions to those were present in the male reproductive tract of BALB/c mice were also present in the male C57BL/6 mice. The segment of the reproductive tract that was most significantly affected was the spermatic cord, with 17/19 (89%) with minimal to marked pyogranulomatous inflammation. Additionally, 15/19 (79%) of all male C57BL/6 mice had necrosis and 7/19 (37%) had abscesses in the spermatic cord. Inflammatory lesions were also present in the epididymis, testes, and accessory sex glands of male C57BL/6 mice at all ages. Mild to severe inflammation was present in the epididymis in 14/19 (74%) mice, with necrosis noted 12/19 (63%) and abscess formation in 6/19 (31%) male C57BL/6 mice. Minimal to marked inflammation was present in the tunic of the testes in 10/19 (52%) of all male mice, with the inflammation extending into the interstitium resulting in necrosis of the seminiferous tubules in 2/19 (10%) of the mice. Varying degrees of inflammation were noted within the accessory sex glands (prostate gland, seminal vesicles, and coagulating gland) of male C57BL/6 mice.

**Figure 4:**
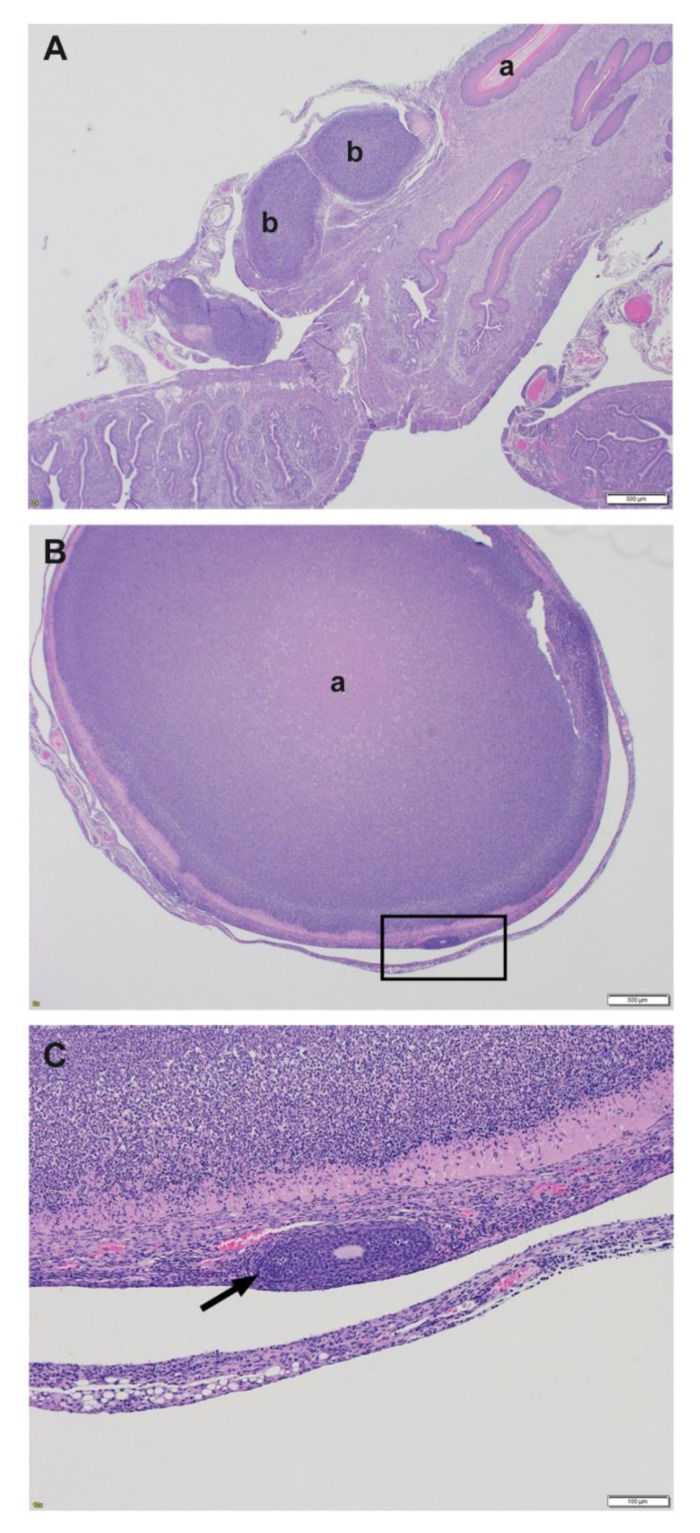
Female reproductive tract pathology associated with intraperitoneal exposure to *B. pseudomallei*. (**A**) Uterine serosa with moderate pyogranulomatous inflammation, necrosis, and abscesses (BALB/c mouse 28 weeks at challenge, H&E); a: uterine lumen, b: abscess (**B**) Ovary with locally extensive abscess (C57BL/6 18 weeks at challenge, H&E); a: abscess. (**C**) Magnification of black box identified in 5B demonstrating moderate pyogranulomatous inflammation with extensive abscess (arrow points to a single remaining primary follicle in the ovary) (C57BL/6 mouse 18 weeks at challenge, H&E).

Significant histological findings in the reproductive tract of female BALB/c mice were present in the uterus. Moderate pyogranulomatous inflammation with necrosis and abscess formation in the broad ligament and serosal surface was present in 3/22 (14%) females; all 3 affected females were 28 weeks old at challenge (Figure 4A). Additionally 2/22 (9%) females had mild pyogranulomatous inflammation in the periovarian connective tissue.

Histological findings in the reproductive tract of female C57BL/6 mice were present in the uterus and ovary. Moderate pyogranulomatous inflammation with necrosis and abscesses were present on the uterine serosal surface in 2/13 (15%) females, both of which were 28 weeks old. Additionally, 1/13 (7%) females had mild to moderate pyogranulomatous inflammation in the periovarian connective tissue and 3/13 (23%) females had a unilateral locally extensive abscess that replaced 80%–98% cortical stroma and medulla of the ovary with moderate pyogranulomatous inflammation in the remaining ovarian and surrounding connective tissue (Figure 4B,C).

#### 2.4.2. Inhalational Route of Infection

The percentage of mice exhibiting significant peritonitis was appreciably lower in mice exposed to aerosolized bacteria. Mild to marked inflammation in the peritoneum was present in 11/33 (33%) of the male BALB/c mice. Mild to marked inflammation in the peritoneum was present in 10/58 (17%) of the female BALB/c mice. Marked inflammation in the peritoneum was present in 1/17 (6%) of the male C57BL/6 mice. Minimal to mild inflammation in the peritoneum was present in 2/24 (8%) of the female C57BL/6 mice.

The segment of the reproductive tract that was most commonly affected in male BALB/c mice infected via the inhalational route was the spermatic cord, with 4/33 (12%) with minimal to moderate pyogranulomatous inflammation and necrosis with abscesses noted in 1/33 (3%) of the male mice. Mild inflammation was noted in testicular capsule of 1/33 (3%) and in the smooth muscle surrounding the epididymis in 2/33 (6%) of the male mice. In the accessory sex glands, mild inflammation was present in the seminal vesicles in 2/33 (6%), and minimal to mild inflammation was present in the prostate gland in 3/33 (9%) of the male mice. The coagulating gland was unremarkable in all of the male mice examined.

The segment of the reproductive tract that was most commonly affected in male C57BL/6 mice infected after exposure to aerosolized bacteria was the spermatic cord, with 2/17 (12%) with mild to moderate pyogranulomatous inflammation and necrosis. All examined testes and epididymis were unremarkable. The prostate was the only affected accessory sex gland; the affected mouse 1/17 (6%) had mild inflammation in the connective tissue surrounding the gland.

Inflammation in the reproductive tract of female BALB/c mice was present only in the periovarian connective tissue of 1/49 (2%) female BALB/c mouse (ovaries for nine female BALB/c mice were not present on slides). No inflammatory lesions were noted in the uterus in 57 BALB/c females examined (uterus for one female BALB/c mouse was not present on the slide). There were no inflammatory lesions present in the reproductive tract of the 24 female C57BL/6 mice examined. 

## 3. Discussion

Our data demonstrated several important nuances associated with the mouse models of melioidosis. The majority of human cases of *B. pseudomallei* infection have been documented in males. This is likely due to occupational and lifestyle differences between males and females in endemic regions. However, because of the chronic and insidious nature of this disease, it is possible that anatomical differences could lead to distinct pathogenesis. For example, numerous reports have demonstrated prostatic involvement in human male melioidosis patients [38,68,69,70,71] but few reports exist describing female-specific pathology [72]. Additionally, when placing *B. pseudomallei* in context of protecting the Warfighter, it becomes essential to understand both female and male models of the disease. To our knowledge, this is one of the first reports documenting the pathology observed in the reproductive tracts of mice.

The spermatic cord was the most consistently affected organ in both male BALB/c and C57BL/6 mice challenged intraperitoneally, with pyogranulomatous inflammation, necrosis, and abscesses being the most common histological findings. Inflammatory lesions were also present with a variable percentage in the epididymis, testes, and accessory sex organs. Minimal to mild lesions often started in the capsule/tunic of the testes and epididymis, and with increased severity the inflammation extended into the interstitium of the reproductive organ. The inflammation in the male reproductive tract was likely the result of the route of challenge and not a targeting of the reproductive organs by the bacterium. In general, male mice have large redundant testes that readily retract into the abdominal cavity through their open inguinal rings, especially when they are picked up by the tail [73]. It is possible, given the challenge route and anatomy, that the reproductive tract was directly inoculated, which led to inflammation in the spermatic cord in 100% of BALB/c male mice and in 89% of C57BL/6 male mice. Another possibility is that the male reproductive tract was affected due to local extension of the inflammation from the abdomen through the open inguinal rings. A mild to moderate peritonitis was present in 61% of male BALB/c mice and in 52% of the male C57BL/6 mice. In the early stages of inflammation, the serosal surfaces were affected more often than the interstitial tissue, indicating that the inflammation in the reproductive tract was simply due to local extension. The reproductive tracts of female BALB/c and C57BL/6 mice challenged intraperitoneally did not have any consistent histological findings. The most common finding was pyogranulomatous inflammation, necrosis, and abscesses on the serosal surface of the uterus or in the broad ligament. Similarly to the males that were intraperitoneally challenged, the inflammation was likely the result of local extension, as 81% of BALB/c and 53% of C57BL/6 females had peritonitis. These observations were in stark contrast with those made when examining mice exposed to aerosolized bacteria. Peritonitis in these mice ranged from 5% to 33%; not surprisingly, the reproductive tracts of these mice were less affected.

During the acute phase of the disease (through Day 21), there were no statistically significant differences associated with mouse sex between the LD_50_ equivalents calculated for the different mouse-strain- and infection-route-matched data sets. However, when examining the data through Day 60, female mice were shown to have a significantly greater LD_50_. Perhaps these data suggest that the bacteria disseminate differently in male and female mice, resulting in differences in the more protracted or chronic form of the disease. In addition to anatomical differences, it is also imperative to discuss differences associated with hormone levels and social interactions (e.g., aggression). It is well known that male mice are considerably more aggressive than female mice, and this fact was even more pronounced when we examined C57BL/6 male mice [74,75]. These social interactions often resulted in aggressive behavior and the mice exhibiting signs of increased stress levels [36]. In some cases, mice had to be singly housed in order to avoid physical injury to their less aggressive counterparts. Lastly, as the testes became infected, self-mutilation became apparent, and in some cases resulted in early endpoint euthanasia. These issues made the experimental logistics challenging, but may also have impacted the overall disease outcome, since stress is known to modulate many aspects of the immune response. Nevertheless, in order to have the most complete dataset possible, male mice should be considered.

An equally important variable that has not been examined adequately in the literature is the age of the experimental animals. Because of its chronic nature, *B. pseudomallei* can often become reactivated in older individuals that may have been infected much earlier in their life. Additionally, when designing experimental vaccine regimens, the mice will be substantially older at the time of challenge with *B. pseudomallei* when compared to the vaccination. Thus, it may not be appropriate to use the same data generated using 8–10 week old mice when designing vaccine trials. Our data illustrated that older mice are significantly more susceptible to the ensuing infection, regardless of route of infection. While the mice used in our study were still considered relatively young, advanced age is known to result in diminished immune responses in both humans and mice [76,77,78,79]. Lastly, we performed limited retrospective statistical analyses comparing current data with historical experiments performed at USAMRIID. We were encouraged and somewhat surprised by the relative lack of significant differences observed in these analyses. Perhaps the inherent heterogeneity of mouse models of melioidosis minimizes any differences associated with source vendor that would normally be significant in more acute and homogenous bacterial diseases (e.g., plague or anthrax). 

Age and sex differences have been observed in murine models used for other infectious diseases and high-consequence pathogens. While these differences can vary based upon strain of mouse or isolate/strain of pathogen, the importance of these findings is undeniable. Ren et al. demonstrated that aged mice are more susceptible to *Salmonella typhimurium*, and that this susceptibility appeared to correlate with the impairment of cytokine expression (IFN-γ and TNF-α) and leukocyte response [80]. Shin et al. reported similar data using murine infection models of *Clostridum difficile*, and also hypothesized that this altered susceptibility to disease could be attributed, at least to some extent, to altered innate immunity associated with microbiota-dependent changes associated with age [81,82]. There have been several reports indicating a sex bias associated with *Yersinia pestis* susceptibility, with female mice being more resistant to infection [83,84,85]. Additionally, Lambert and coworkers demonstrated via several parameters that older mice are also more susceptible to plague disease compared to younger mice [84]. Using the *Francisella* live vaccine strain as a surrogate pathogen, Mares et al. identified no significant differences associated with age and survival; they did, however, note several important differences that were impacted in aged mice, including polymorphonuclear neutrophil infiltration kinetics and cytokine expression levels [86]. Interestingly, this work suggested that in addition to the altered immune kinetics associated with aging, lungs of aged control mice were demonstrably more damaged than young control mice. This lung damage likely contributed to the more severe pathology observed when aged mice are infected. While the above examples demonstrate sex and/or age bias with bacterial diseases, other reports suggest little to no impact of these factors on disease. For example, Lyons et al. reported no differences associated with either age or sex in murine models of pulmonary anthrax [87].

Similar differences have also been observed in viral infections. Aged mice have been demonstrated to exhibit a more severe disease after exposure to respiratory syncytial virus (RSV) compared to their younger counterparts [88,89]. The aged mice demonstrated enhanced pathology, altered antiviral gene expression, and a diminished CD8 T cell response [88,89]. Recently, it has also been demonstrated that there are sex-associated differences in RSV infection in neonatal mice. Malinczak et al. described an altered response in male neonates which ultimately led to a more severe allergic response later in life [90]. Older mice have reduced natural killer cell population and function, which have been linked with increased susceptibility to mousepox virus [91]. However, mouse parvovirus has been shown to be readily transmitted to mice regardless of the age of the mice tested [92,93]. Importantly, there are numerous viruses that rely on neonatal/suckling mice to model disease, as older mice exhibit less appreciable clinical signs of illness or subsequent mortality rates. This is exemplified in several alphavirus and flavivirus models [94,95,96].

In summary, these data underscore the complexities of designing adequate studies for testing novel medical countermeasures in mice. This is particularly the case for models of melioidosis due to significant heterogeneity of clinical signs and the varying levels of chronicity associated with different strains of mice. In addition to selecting the mouse strain and bacterial isolate to be tested, the sex, age, and source of the experimental animals must also be considered in order to generate the most robust model system possible.

## 4. Materials and Methods 

### 4.1. Animal Challenges

Groups of BALB/c or C57BL/6 mice (Envigo, Buckeystown, MD, USA), female or male and either 8, 18, or 28 weeks of age at time of exposure to bacteria were challenged by IP or inhalational route with a low passage and well-defined stock of *B. pseudomallei* K96243 [25,27]. Most groups contained 10 mice, but, due to aggressive behavior, some groups of male mice contained 7–9 mice. Where indicated, historical data collected from mice obtained from other vendors were used for statistical comparisons (Charles River, Frederick, MD, USA and JAX Labs, Bar Harbor, ME, USA). The bacteria used were grown in 4% glycerol (Sigma Aldrich, St. Louis, MO, USA) 1% tryptone (Difco, Becton Dickinson, Sparks, MD, USA), and 5% NaCl (Sigma Aldrich, St. Louis, MO, USA) broth (GTB) at 37 °C with shaking at 200 rpm, and were harvested from a late log-phase culture. The bacteria were resuspended in GTB and quantified via OD_620_ estimations. The actual delivered doses of bacteria were then verified by plate counts on sheep’s blood agar (SBA) plates (Remel, ThermoFisher Scientific, Waltham, MA, USA). Each IP dose was delivered in 200 μl of GTB medium. Exposure to aerosolized bacteria was accomplished as previously described [97]. Briefly, mice were transferred to wire mesh cages (up to 10 mice per cage), and up to four wire mesh cages were placed in a whole-body aerosol chamber within a class three biological safety cabinet located inside a BSL-3 laboratory. Mice were exposed to aerosolized *B. pseudomallei* strain K96243 created by a three-jet collision nebulizer. Samples were collected from the all-glass impinger (AGI) and analyzed by performing CFU calculations to determine the inhaled dose of *B. pseudomallei.*

### 4.2. Histological Pathology

Post-mortem tissues were collected from representative euthanized mice and fixed in 10% neutral buffered formalin for ≥21 days [98]. Samples were embedded in paraffin and sectioned for hematoxylin and eosin (HE) staining, as previously described [25,99].

### 4.3. Ethics Statement

Animal research at the United States Army Medical Research Institute of Infectious Diseases (USAMRIID) was conducted under an animal use protocol approved by the USAMRIID Institutional Animal Care and Use Committee (IACUC) in compliance with the Animal Welfare Act, PHS Policy, and other Federal statutes and regulations relating to animals and experiments involving animals. The facility where this research was conducted is accredited by the Association for Assessment and Accreditation of Laboratory Animal Care International (AAALACi) and adheres to the principles set down in the Guide for the Care and Use of Laboratory Animals (National Research Council, 2011). Challenged mice were observed at least daily for up to 60 days for clinical signs of illness. Early intervention endpoints were used during all studies and mice were euthanized when moribund, according to an endpoint score sheet. Animals were scored on a scale of 0–11: 0–2 = no significant clinical signs (e.g., slightly ruffled fur); 3–7 = significant clinical symptoms such as subdued behavior, hunched appearance, absence of grooming, hind-limb issues of varying severity and/or pyogranulomatous swelling of varying severity (increased monitoring was warranted); 8–11 = distress. Those animals receiving a score of 8–11 were euthanized. However, even with multiple observations per day, some animals died as a direct result of the infection.

### 4.4. Statistical Analyses

LD_50_s were estimated by probit regression, with confidence limits by Fieller’s method [100]. LD_50_s were compared among age and sex categories by probit regression, under the assumption of a common probit slope. The effect of age was expressed as the reduction in Log_10_ LD_50_ relative to animals aged 8 weeks, and estimates of the relative potencies were averaged across sexes as described by Finney [101]. The effect of sex was similarly pooled across age strata, where appropriate. Confidence intervals on the relative potencies were obtained via the delta method. The Wald chi-square test was used to determine the statistical significance of regression parameters. The analysis was implemented in SAS PROC/PROBIT and PROC/GENMOD, SAS version 9.4 (SAS Institute, Cary, NC, USA).

## Figures and Tables

**Table 1:** The calculated median lethal dose of *B. pseudomallei* K96243 in female and male BALB/c and C57BL/6 mice in three age groups ^1^.

A. Day 21 LD_50_				Age at Exposure (Weeks)		
*Gender*	*Exposure* *Route*	*Mouse* *Strain*	*Dose Range* *Min, Max*	*8*	*18*	*28*
Female	Aerosol	BALB/c	−0.16 to 4.00	0.84 (0.36, 1.32)	0.18 (−0.46, 0.64)	−0.16 (−11.14, 0.19)
		C57BL/6	−0.19 to 4.02	2.67 (− ^2^)	2.78 (2.31, 3.40)	1.97 (1.51, 2.52)
	IP	BALB/c	1.83 to 5.66	4.04 (3.77, 4.29)	3.74 (3.18, 4.37)	2.86 (1.04, 3.71)
		C57BL/6	4.11 to 8.07	5.79 (5.32, 6.24)	6.27 (5.86, 6.70)	5.19 (− ^2^)
Male	Aerosol	BALB/c	−0.18 to 3.26	0.48 (0.00, 0.94)	0.32 (0.04, 0.73)	−0.58 (− ^2^)
		C57BL/6	0.10 to 4.05	3.02 (2.51, 3.77)	2.19 (− ^2^)	2.28 (1.84, 2.80)
	IP	BALB/c	2.16 to 6.09	4.48 (4.05, 4.95)	3.55 (2.84, 4.16)	2.26 (−38.27, 3.47)
		C57BL/6	3.93 to 8.00	6.37 (5.87, 6.95)	5.25 (4.42, 5.93)	4.48 (1.48, 5.38)
**B. Day 60 LD_50_**						
Female	Aerosol	BALB/c	−0.16 to 4.00	0.00 (−1.49, 0.41)	−0.22 (− ^2^)	−0.32 (− ^2^)
		C57BL/6	−0.19 to 4.02	2.62 (− ^2^)	2.67 (2.22, 3.26)	1.55 (1.15, 2.05)
	IP	BALB/c	1.83 to 5.66	3.02 (2.02, 3.68)	−6.47 (− ^2^)	1.83 ^3^
		C57BL/6	4.11 to 8.07	5.51 (5.07, 5.92)	5.56 (5.07, 6.04)	4.07 (− ^2^)
Male	Aerosol	BALB/c	−0.18 to 3.26	−0.22 (−19.85, 0.14)	−0.43 (− ^2^)	−0.89 (− ^2^)
		C57BL/6	0.10 to 4.05	2.92 (2.42, 3.60)	1.96 (1.36, 2.63)	2.10 (1.63, 2.63)
	IP	BALB/c	2.16 to 6.09	2.16 ^3^	1.95 (− ^2^)	2.16 ^3^
		C57BL/6	3.93 to 8.00	5.42 (4.63, 6.12)	3.82 (−2.89, 4.59)	3.93 ^3^

^1^ Values indicate LD_50_ in Log_10_(CFU) and 95% confidence interval. ^2^ Unbounded confidence intervals. ^3^ No survivors remained, the least challenge dose is reported.

**Table 2:** Impact of source vendor on the disease outcome ^1^.

.				Age at Exposure (Weeks)		
*Gender*	*Exposure* *Route*	*Source* *Vendor*	*Mouse* *Strain*	*8*	*18*	*28*
Female	Aerosol	Charles River	BALB/c	*p* > 0.05	ND	ND
			C57BL/6	*p* > 0.05	ND	ND
	IP	Charles River	BALB/c	*p* > 0.05	ND	ND
			C57BL/6	*p* = 0.037	ND	ND
Male	IP	Jackson Laboratory	C57BL/6	*p* > 0.05	*p* > 0.05	*p* > 0.05

^1^*p*-value calculated comparing mice from this vendor with mice from Envigo.

## References

[1] Dance D.A. (1991). Melioidosis: The tip of the iceberg?. Clin. Microbiol. Rev..

[2] Dance D.A. (2000). Melioidosis as an emerging global problem. Acta Trop..

[3] Limmathurotsakul D., Golding N., Dance D.A., Messina J.P., Pigott D.M., Moyes C.L., Rolim D.B., Bertherat E., Day N.P., Peacock S.J. (2016). Predicted global distribution of and burden of melioidosis. Nat. Microbiol..

[4] Hassan M.R., Pani S.P., Peng N.P., Voralu K., Vijayalakshmi N., Mehanderkar R., Aziz N.A., Michael E. (2010). Incidence, risk factors and clinical epidemiology of melioidosis: A complex socio-ecological emerging infectious disease in the Alor Setar region of Kedah, Malaysia. BMC Infect. Dis..

[5] Thomas R.J., Davies C., Nunez A., Hibbs S., Eastaugh L., Harding S., Jordan J., Barnes K., Oyston P., Eley S. (2012). Particle-size dependent effects in the BALB/c murine model of inhalational melioidosis. Front. Cell. Infect. Microbiol..

[6] West T.E., Myers N.D., Liggitt H.D., Skerrett S.J. (2012). Murine pulmonary infection and inflammation induced by inhalation of *Burkholderia pseudomallei*. Int. J. Exp. Pathol..

[7] Limmathurotsakul D., Wongsuvan G., Aanensen D., Ngamwilai S., Saiprom N., Rongkard P., Thaipadungpanit J., Kanoksil M., Chantratita N., Day N.P.J. (2014). Melioidosis caused by *Burkholderia pseudomallei* in drinking water, Thailand, 2012. Emerg. Infect. Dis..

[8] Suputtamongkol Y., Chaowagul W., Chetchotisakd P., Lertpatanasuwun N., Intaranongpai S., Ruchutrakool T., Budhsarawong D., Mootsikapun P., Wuthiekanun V., Teerawatasook N. (1999). Risk factors for melioidosis and bacteremic melioidosis. Clin. Infect. Dis..

[9] Amemiya K., Bozue J.A., Cote C.K., DeShazer D., Soffler C., Welkos S.L., Worsham P.L. (2017). Animal models for melioidosis. Curr. Trop. Med. Rep..

[10] Currie B.J., Jacups S.P. (2003). Intensity of rainfall and severity of melioidosis, Australia. Emerg. Infect. Dis..

[11] Liu X., Pang L., Sim S.H., Goh K.T., Ravikumar S., Win M.S., Tan G., Cook A.R., Fisher D., Chai L.Y. (2015). Association of melioidosis incidence with rainfall and humidity, Singapore, 2003-2012. Emerg. Infect. Dis..

[12] Meumann E.M., Cheng A.C., Ward L., Currie B.J. (2012). Clinical features and epidemiology of melioidosis pneumonia: Results from a 21-year study and review of the literature. Clin. Infect. Dis..

[13] Currie B.J. (2003). Melioidosis: An important cause of pneumonia in residents of and travellers returned from endemic regions. Eur. Respir. J..

[14] Dance D. (2014). Treatment and prophylaxis of melioidosis. Int. J. Antimicrob. Agents.

[15] Limmathurotsakul D., Funnell S.G., Torres A.G., Morici L.A., Brett P.J., Dunachie S., Atkins T., Altmann T.M., Bancroft G., Peacock S.J. (2015). Consensus on the development of vaccines against naturally acquired melioidosis. Emerg. Infect. Dis..

[16] Peacock S.J., Limmathurotsakul D., Lubell Y., Koh G.C., White L.J., Day N.P., Titball R.W. (2012). Melioidosis vaccines: A systematic review and appraisal of the potential to exploit biodefense vaccines for public health purposes. PLoS Negl. Trop. Dis..

[17] Barnes J.L., Ketheesan N. (2005). Route of infection in melioidosis. Emerg. Infect. Dis..

[18] Barnes J.L., Ulett G.C., Ketheesan N., Clair T., Summers P.M., Hirst R.G. (2001). Induction of multiple chemokine and colony-stimulating factor genes in experimental *Burkholderia pseudomallei* infection. Immunol. Cell Biol..

[19] Barnes J.L., Warner J., Melrose W., Durrheim D., Speare R., Reeder J.C., Ketheesan N. (2004). Adaptive immunity in melioidosis: A possible role for T cells in determining outcome of infection with *Burkholderia pseudomallei*. Clin. Immunol..

[20] Barnes J.L., Williams N.L., Ketheesan N. (2008). Susceptibility to *Burkholderia pseudomallei* is associated with host immune responses involving tumor necrosis factor receptor-1 (TNFR1) and TNF receptor-2 (TNFR2). FEMS Immunol. Med. Microbiol..

[21] Leakey A.K., Ulett G.C., Hirst R.G. (1998). BALB/c and C57Bl/6 mice infected with virulent *Burkholderia pseudomallei* provide contrasting animal models for the acute and chronic forms of human melioidosis. Microb. Pathog..

[22] Bearss J.J., Hunter M., Dankmeyer J.L., Fritts K.A., Klimko C.P., Weaver C.H., Shoe J.L., Quirk A.V., Toothman R.G., Webster W.M. (2017). Characterization of pathogenesis of and immune response to *Burkholderia pseudomallei* K96243 using both inhalational and intraperitoneal infection models in BALB/c and C57BL/6 mice. PLoS ONE.

[23] Tan G.Y., Liu Y., Sivalingam S.P., Sim S.H., Wang D., Paucod J.C., Gathier Y., Ooi E.E. (2008). *Burkholderia pseudomallei* aerosol infection results in differential inflammatory responses in BALB/c and C57Bl/6 mice. J. Med. Microbiol..

[24] Trevino S.R., Klimko C.P., Reed M.C., Aponte-Cuadrado M.J., Hunter M., Shoe J.L., Meyer J.R., Dankmeyer J.L., Biryukov S.S., Quirk A.V. (2018). Disease progression in mice exposed to low-doses of aerosolized clinical isolates of *Burkholderia pseudomallei*. PLoS ONE.

[25] Welkos S.L., Klimko C.P., Kern S.J., Bearss J.J., Bozue J., Bernhards R.C., Trevin S., Waag D.M., Amemiya K., Worsham P. (2015). Characterization of *Burkholderia pseudomallei* strains using a murine intraperitoneal infection model and in vitro macrophage assays. PLoS ONE.

[26] Amemiya K., Dankmeyer J.L., Fetterer D.P., Worsham P.L., Welkos S.L., Cote C.K. (2015). Comparison of the early host immune response to two widely diverse virulent strains of *Burkholderia pseudomallei* that cause acute and chronic infections in BALB/c mice. Microb. Pathog..

[27] Van Zandt K.E., Tuanyok A., Keim P.S., Warren R.L., Gelhaus H.C. (2012). An objective approach for *Burkholderia pseudomallei* strain selection as challenge material for medical countermeasures efficacy testing. Front. Cell. Infect. Microbiol..

[28] Dannenberg A.M., Scott E.M. (1958). Melioidosis: Pathogenesis and immunity in mice and hamsters. I. Studies with virulent strains of *Malleomyces pseudomallei*. J. Exp. Med..

[29] Raja N.S., Scarsbrook C. (2016). *Burkholderia Pseudomallei* Causing Bone and Joint Infections: A Clinical Update. Infect. Dis. Ther..

[30] Teparrakkul P., Tsai J.J., Chierakul W., Gerstenmaier J.F., Wacharaprechasgu T., Piyaphanee W., Limmathurotsakul D., Chaowagul W., Day N.P., Peacock S.J. (2008). Rheumatological manifestations in patients with melioidosis. Southeast Asian J. Trop. Med. Public Health.

[31] Chen P.S., Chen Y.S., Lin H.H., Liu P.J., Ni W.F., Hsueh P.T., Liang S.H., Chen C., Chen Y.L. (2015). Airborne transmission of melioidosis to humans from environmental aerosols contaminated with *B. pseudomallei*. PLoS Negl. Trop. Dis..

[32] Lever M.S., Nelson M., Stagg A.J., Beedham R.J., Simpson A.J. (2009). Experimental acute respiratory *Burkholderia pseudomallei* infection in BALB/c mice. Int. J. Exp. Pathol..

[33] Massey S., Yeager L.A., Blumentritt C.A., Vijayakumar S., Sbrana E., Peterson J.W., Brasel T., LeDuc J.W., Endsley J.J., Torres A.G. (2014). Comparative *Burkholderia pseudomallei* natural history virulence studies using an aerosol murine model of infection. Sci. Rep..

[34] Challacombe J.F., Stubben C.J., Klimko C.P., Welkos S.L., Kern S.J., Bozue J.A., Worsham P.L., Cote C.K., Wolfe D.L. (2014). Interrogation of the *Burkholderia pseudomallei* Genome to Address Differential Virulence among Isolates. PLoS ONE.

[35] Hoppe I., Brenneke B., Rohde M., Kreft A., Häußler S., Reganzerowski A., Steinmetz I. (1999). Characterization of a murine model of melioidosis: Comparison of different strains of mice. Infect. Immun..

[36] Webster Marketon J.I., Glaser R. (2008). Stress hormones and immune function. Cell. Immunol..

[37] Corea E.M., Merritt A.J., Ler Y.H., Thevanesam V., Inglis T.J. (2016). Sri Lankan National Melioidosis surveillance program uncovers a nationwide distribution of invasive melioidosis. Am. J. Trop. Med. Hyg..

[38] Currie B.J., Jacups S.P., Cheng A.C., Fisher D.A., Anstey N.M., Huffam S.E., Krause V.L. (2004). Melioidosis epidemiology and risk factors from a prospective whole-population study in northern Australia. Trop. Med. Int. Health.

[39] Suntornsut P., Kasemsupat K., Silairatana S., Wongsuvan G., Jutrakul Y., Wuthiekanun V., Day N.P., Peacock S.J., Limmathurotsakul D. (2013). Prevalence of melioidosis in patients with suspected pulmonary tuberculosis and sputum smear negative for acid-fast bacilli in northeast Thailand. Am. J. Trop. Med. Hyg..

[40] Klein S.L., Flanagan K.L. (2016). Sex differences in immune responses. Nat. Rev. Immunol..

[41] Chirakul S., Bartpho T., Wongsurawat T., Taweechaisupapong S., Karoonutaisiri N., Talaat A.M., Wongratanacheewin S., Ernst R.K., Sermswan R.W. (2014). Characterization of BPSS1521 (*bprD*), a regulator of *Burkholderia pseudomallei* virulence gene expression in the mouse model. PLoS ONE.

[42] Gelhaus H.C., Anderson M.S., Fisher D.A., Flavin M.T., Xu Z.Q., Sanford D.C. (2013). Efficacy of post exposure administration of doxycycline in a murine model of inhalational melioidosis. Sci. Rep..

[43] Ulett G.C., Ketheesan N., Hirst R.G. (2000). Cytokine gene expression in innately susceptible BALB/c mice and relatively resistant C57BL/6 mice during infection with virulent *Burkholderia pseudomallei*. Infect. Immun..

[44] Wiersinga W.J., Calandra T., Kager L.M., van der Windt G.J., Roger T., le Roy D., Florquin S., Peacock S.J., Sweep F.C., van der Poll T. (2010). Expression and function of macrophage migration inhibitory factor (MIF) in melioidosis. PLoS Negl. Trop. Dis..

[45] Hodgson K.A., Govan B.L., Walduck A.K., Ketheesan N., Morris J.L. (2013). Impaired early cytokine responses at the site of infection in a murine model of type 2 diabetes and melioidosis comorbidity. Infect. Immun..

[46] Puangpetch A., Anderson R., Huang Y.Y., Sermswan R.W., Chaicumpa W., Sirisinha S., Wongratanacheewin S. (2012). Cationic liposomes extend the immunostimulatory effect of CpG oligodeoxynucleotide against *Burkholderia pseudomallei* infection in BALB/c mice. Clin. Vaccine Immunol..

[47] Tay T.F., Maheran M., Too S.L., Hasidah M.S., Ismail G., Embi N. (2012). Glycogen synthase kinase-3beta inhibition improved survivability of mice infected with *Burkholderia pseudomallei*. Trop Biomed..

[48] Kager L.M., Weehuizen T.A., Wiersinga W.J., Roelofs J.J., Meijers J.C., Dondorp A.M., van‘t Veer C., van der Poll T. (2013). Endogenous alpha2-antiplasmin is protective during severe gram-negative sepsis (melioidosis). Am. J. Respir. Crit. Care Med..

[49] Kager L.M., Wiersinga W.J., Roelofs J.J., Stroo I., Achouiti A., van’t Veer C., Conway E.M., van der Poll T. (2014). Mice lacking the lectin-like domain of thrombomodulin are protected against melioidosis. Crit. Care Med..

[50] Weehuizen T.A., Wieland C.W., van der Windt G.J., Duitman J.W., Boon L., Day N.P., Peacock S.J., van der Poll T., Wiersinga W.J. (2012). Expression and function of transforming growth factor beta in melioidosis. Infect. Immun..

[51] Mariappan V., Vellasamy K.M., Vadivelu J. (2017). Host-adaptation of *Burkholderia pseudomallei* alters metabolism and virulence: A global proteome analysis. Sci. Rep..

[52] Weehuizen T.A., Prior J.L., van der Vaart T.W., Ngugi S.A., Nepogodiev S.A., Field R.A., Kager L.M., van’t Veer C., de Vos A.F., Wiersinga W.J. (2015). Differential Toll-like receptor-signalling of *Burkholderia pseudomallei* lipopolysaccharide in murine and human models. PLoS ONE.

[53] Emery F.D., Parvathareddy J., Pandey A.K., Cui Y., Williams R.W., Miller M.A. (2014). Genetic control of weight loss during pneumonic *Burkholderia pseudomallei* infection. Pathog. Dis..

[54] Kager L.M., Wiersinga W.J., Roelofs J.J., de Boer O.J., Weiler H., van’t Veer C., van der Poll T. (2014). A thrombomodulin mutation that impairs active protein C generation is detrimental in severe pneumonia-derived gram-negative sepsis (melioidosis). PLoS Negl. Trop. Dis..

[55] Baker D.G. (1998). Natural pathogens of laboratory mice, rats, and rabbits and their effects on research. Clin. Microbiol. Rev..

[56] Kline K.A., Schwartz D.J., Gilbert N.M., Lewis A.L. (2014). Impact of host age and parity on susceptibility to severe urinary tract infection in a murine model. PLoS ONE.

[57] Han S.N., Meydani S.N. (2000). Antioxidants, cytokines, and influenza infection in aged mice and elderly humans. J. Infect. Dis..

[58] Lu J., Duan X., Zhao W., Wang J., Wang H., Zhou K., Fang M. (2018). Aged mice are more resistant to influenza virus infection due to reduced inflammation and lung pathology. Aging Dis..

[59] Pal S., Peterson E.M., de la Maza L.M. (2001). Susceptibility of mice to vaginal infection with *Chlamydia trachomatis* mouse pneumonitis is dependent on the age of the animal. Infect. Immun..

[60] Puchta A., Naidoo A., Verschoor C.P., Loukov D., Thevaranjan N., Mandur T.S., Nguyen P.S., Jordana M., Loeb M., Xing Z. (2016). TNF drives monocyte dysfunction with age and results in impaired anti-pneumococcal immunity. PLoS Pathog..

[61] Zhang Y., Wang Y., Gilmore X., Xu K., Wyde P.R., Mbawuike I.N. (2002). An aged mouse model for RSV infection and diminished CD8+ CTL response. Exp. Biol. Med..

[62] Ngauy V., Lemeshev Y., Sadkowski L., Crawford G. (2005). Cutaneous melioidosis in a man who was taken as a prisoner of war by the Japanese during World War II. J. Clin. Microbiol..

[63] Limmathurotsakul D., Chaowagul W., Chantratita N., Wuthiekanun V., Biaklang M., Tumapa S., White N.J., Day N.P., Peacock S.J. (2008). A simple scoring system to differentiate between relapse and re-infection in patients with recurrent melioidosis. PLoS Negl. Trop. Dis..

[64] Jenjaroen K., Chumseng S., Sumonwiriya M., Ariyaprasert P., Chantratita N., Sunyakumthorn P., Hongsuwan M., Wuthiekanun V., Fletcher H.A., Teparrukkul P. (2015). T-cell responses are associated with survival in acute melioidosis patients. PLoS Negl. Trop. Dis..

[65] Of mice and men—are mice relevant models for human disease?. https://ec.europa.eu/research/health/pdf/summary-report-25082010_en.pdf.

[66] Justice M.J., Dhillon P. (2016). Using the mouse to model human disease: Increasing validity and reproducibility. Dis. Models Mech..

[67] Uhl E.W., Warner N.J. (2015). Mouse models as predictors of human responses: Evolutionary medicine. Curr. Pathobiol. Rep..

[68] Kozlowska J., Smith S., Roberts J., Pridgeon S., Hanson J. (2018). Prostatic abscess due to *Burkholderia pseudomallei*: Facilitating diagnosis to optimize management. Am. J. Trop. Med. Hyg..

[69] Morse L.P., Moller C.C., Harvey E., Ward L., Cheng A.C., Carson P.J., Currie B.J. (2009). Prostatic abscess due to *Burkholderia pseudomallei*: 81 cases from a 19-year prospective melioidosis study. J. Urol..

[70] Currie B.J., Ward L., Cheng A.C. (2010). The epidemiology and clinical spectrum of melioidosis: 540 cases from the 20 year Darwin prospective study. PLoS Negl. Trop. Dis..

[71] Chee Y.C., Chee Y.N. (2018). An unusual case of primary melioidotic prostatic abscess complicated by perianal abscess. IDCases.

[72] Nernsai P., Sophonsritsuk A., Lertvikool S., Jinawath A., Chitasombat M.N. (2018). A case report of Tubo-ovarian abscess caused by *Burkholderia pseudomallei*. BMC Infect. Dis..

[73] Barthold S.W., Griffey S.M., Percy D.H. (2016). Pathology of Laboratory Rodents and Rabbits.

[74] Van Loo P.L.P., Van der Meer E., Kruitwagen C.L.J.J., Koolhaas J.M., Van Zutphen L.F.M., Baumans V. (2003). Strain-specific aggressive behavior of male mice submitted to different husbandry procedures. Aggress. Behav..

[75] Kappel S., Hawkins P., Mendl M.T. (2017). To group or not to group? Good practice for housing male laboratory mice. Animals.

[76] Dorshkind K., Swain S. (2009). Age-associated declines in immune system development and function: Causes, consequences, and reversal. Curr. Opin. Immunol..

[77] Kishimoto S., Tsuyuguchi I., Yamamura Y. (1969). Immune responses in aged mice. Clin. Exp. Immunol..

[78] Montecino-Rodriguez E., Berent-Maoz B., Dorshkind K. (2013). Causes, consequences, and reversal of immune system aging. J. Clin. Investig..

[79] Myers C.E., Mirza N.N., Lustgarten J. (2011). Immunity, cancer and aging: Lessons from mouse models. Aging Dis..

[80] Ren Z., Gay R., Thomas A., Pae M., Wu D., Logsdon L., Mecsas J., Meydani1 S.N. (2009). Effect of age on susceptibility to *Salmonella Typhimurium* infection in C57BL/6 mice. J. Med. Microbiol..

[81] Shin J.H., Gao Y., Moore J.H., Bolick D.T., Kolling G.L., Wu M., Warren C.A. (2018). Innate immune response and outcome of *Clostridium difficile* infection are dependent on fecal bacterial composition in the aged host. J. Infect. Dis..

[82] Fischer N., Relman D.A. (2018). Clostridium difficile, Aging, and the gut: Can microbiome rejuvenation keep us young and healthy?. J. Infect. Dis..

[83] Bowen W., Batra L., Pulsifer A.R., Yolcu E.S., Lawrenz M.B., Shirwan H. (2019). Robust Th1 cellular and humoral responses generated by the *Yersinia pestis* rF1-V subunit vaccine formulated to contain an agonist of the CD137 pathway do not translate into increased protection against pneumonic plague. Vaccine.

[84] Lambert N.D., Langfitt D.M., Nilles M.L., Bradley D.S. (2011). Resistance to *Yersinia pestis* infection decreases with age in B10.T(6R) mice. Infect. Immun..

[85] Mecsas J., Franklin G., Kuziel W.A., Brubaker R.R., Falkow S., Mosier D.E. (2004). Evolutionary genetics: CCR5 mutation and plague protection. Nature.

[86] Mares C.A., Ojeda S.S., Li Q., Morris E.G., Coalson J.J., Teale J.M. (2010). Aged mice display an altered pulmonary host response to *Francisella tularensis* live vaccine strain (LVS) infections. Exp. Gerontol..

[87] Lyons C.R., Lovchik J., Hutt J., Lipscomb M.F., Wang E., Heninger S., Berliba L., Garrison K. (2004). Murine model of pulmonary anthrax: Kinetics of dissemination, histopathology, and mouse strain susceptibility. Infect. Immun..

[88] Fulton R.B., Weiss K.A., Pewe L.L., Harty J.T., Varga S.M. (2013). Aged mice exhibit a severely diminished CD8 T cell response following respiratory syncytial virus infection. J. Virol..

[89] Wong T.M., Boyapalle S., Sampayo V., Nguyen H.D., Bedi R., Kamath S.G., Moore M.L., Mohapatra S., Mohapatra S.S. (2014). Respiratory syncytial virus (RSV) infection in elderly mice results in altered antiviral gene expression and enhanced pathology. PLoS ONE.

[90] Malinczak C.A., Fonseca W., Rasky A.J., Ptaschinski C., Morris S., Ziegler S.F., Lukacs N.W. (2019). Sex-associated TSLP-induced immune alterations following early-life RSV infection leads to enhanced allergic disease. Mucosal Immunol..

[91] Fang M., Roscoe F., Sigal L.J. (2010). Age-dependent susceptibility to a viral disease due to decreased natural killer cell numbers and trafficking. J. Exp. Med..

[92] Grove K.A., Smith P.C., Booth C.J., Compton S.R. (2012). Age-associated variability in susceptibility of Swiss Webster mice to MPV and other excluded murine pathogens. J. Am. Assoc. Lab. Anim. Sci..

[93] Compton S.R., Paturzo F.X., Macy J.D. (2012). Transmission of mouse parvovirus to neonatoal mice. J. Am. Assoc. Lab. Anim. Sci..

[94] Blazquez A.B., Escribano-Romero E., Martin-Acebes M.A., Petrovic T., Saiz J.C. (2015). Limited susceptibility of mice to Usutu virus (USUV) infection and induction of flavivirus cross-protective immunity. Virology.

[95] de Oliveira Souza I.N., Frost P.S., Franca J.V., Nascimento-Viana J.B., Neris R.L.S., Freitas L., Pinheiro D.J.L.L., Noqueira C.O., Neves G., Chimelli L. (2018). Acute and chronic neurological consequences of early-life Zika virus infection in mice. Sci. Transl. Med..

[96] Ryman K.D., Gardner C.L., Meier K.C., Biron C.A., Johnston R.E., Klimstra W.B. (2007). Early restriction of alphavirus replication and dissemination contributes to age-dependent attenuation of systemic hyperinflammatory disease. J. Gen. Virol..

[97] Zumbrun E.E., Abdeltawab N.F., Bloomfield H.A., Chance T.B., Nichols D.K., Harrison P.E., Kotb M., Nalca A. (2012). Development of a murine model for aerosolized ebolavirus infection using a panel of recombinant inbred mice. Viruses.

[98] Chua J., Bozue J.A., Klimko C.P., Shoe J.L., Ruiz S.I., Jensen C.L., Tobery S.A., Crumpler J.M., Chabot D.J., Quirk A.V. (2019). Formaldehyde and glutaraldehyde inactivation of bacterial Tier 1 select agents in tissues. Emerg. Infect. Dis..

[99] Davis K.J., Vogel P., Fritz D.L., Steele K.E., Pitt M.L., Welkos S.L., Friedlander A.M., Byrne W.R. (1997). Bacterial filamentation of *Yersinia pestis* by beta-lactam antibiotics in experimentally infected mice. Arch. Pathol. Lab. Med..

[100] Hirschberg J., Lye J. (2010). A geometric comparison of the delta and Fieller confidence intervals. Am. Stat..

[101] Finney D.J. (1971). Probit Analysis.

